# A case report of renal cyst communication regression secondary to hydroureteronephrosis decompression

**DOI:** 10.1016/j.radcr.2026.01.036

**Published:** 2026-02-02

**Authors:** Jerry Yue, Nathan Zhang, Matthew Nwerem, Jesse Kao, Stuart Deaderick, Hardeep Singh

**Affiliations:** a Transitional Year, Northeast Georgia Medical Center, 743 Spring Street NE, Gainesville, GA 30501, USA; b Morehouse School of Medicine, 720 Westview Dr SW, Atlanta, GA 30310, USA; c Philadelphia College of Osteopathic Medicine Georgia, 625 Old Peachtree Rd NW, Suwanee, GA 30024, USA; d Diagnostic Radiology, Northeast Georgia Medical Center, 743 Spring Street NE, Gainesville, GA 30501, USA; e Graduate Medical Education Research, Northeast Georgia Medical Center, 743 Spring Street NE, Gainesville, GA 30501, USA

**Keywords:** Renal, Cyst, Communication, Regression, hydronephrosis, Urinoma

## Abstract

Renal cysts, which are commonly detected incidentally on imaging, often remain asymptomatic. A small percentage may lead to complications such as hemorrhage, infection, or mass effect. This can cause elevated intrapelvic pressure and possible spontaneous rupture, which can establish a communication with the calyceal system. This report presents the case of a 79-year-old male with urothelial carcinoma who developed right-sided hydronephrosis secondary to obstructive malignancy. CT and fluoroscopic imaging revealed a large urinoma with a communication between the collecting system and adjacent renal cyst. Following nephrostomy tube decompression, the presence of contrast within the renal cyst that helped establish the initial communication disappeared and was unable to be re-established. This disappearance contrasts the continued communication seen with typical renal cyst communications and may have occurred due to possible pressure related mechanisms.

## Background

With an estimated prevalence of 27%, simple renal cysts, or fluid-filled pockets that originate from the surface of the kidneys, are commonly detected incidentally through CT or ultrasound [1,2]. Though their exact cause is unclear, their incidence increases with age and male sex [1]. It is estimated that about 25% of individuals over the age of 40, and about 50% of those over the age of 50, have simple kidney cysts [1]. Additionally, the prevalence of renal cysts was 34% in men compared to 21% in women [2].

Most simple renal cysts are typically asymptomatic and require no intervention; however, complications like hemorrhage, infection, or rupture can occur in 2-4% of cases following trauma, enlargement, or bleeding [3]. Occasionally, cysts can become large enough to have a mass effect on other organs or even obstruct urine flow, in which they require interventions like aspiration and sclerotherapy or surgical excision [1]. This potential for urinary obstruction is important to consider in the development of hydronephrosis, the dilation and expansion of the renal collecting system due to blockage of urine flow distal to the renal pelvis [4].

Hydronephrosis, which can also occur due to malignancy or ureteral strictures, can increase intrapelvic pressure and lead to eventual calyceal rupture [4,5]. Though rare, this can lead to the formation of a urinoma, or collections of urine that have leaked outside of the kidney, or other complications like infection, abscess formations, or impaired renal activity [5]. Symptoms of urinomas can range from being asymptomatic to having malaise, pain, hematuria, and changes in urine output [6]. Most urinomas will regress without intervention; however, in more severe cases, they can be treated with a drainage catheter, percutaneous nephrostomy, or placement of a stent across a ureteral defect [6].

Occasionally, renal cysts have also been shown to contribute to hydronephrosis due to their compression of the collecting system [4,7]. Though this relationship between renal cysts and external compression of the pyelocaliceal system has been established, the direct communication between these two has limited literature, and there is still a gap in the literature examining this relationship. Here we present a case where communication between a renal cyst and the collecting system, potentially by renal calyx rupture or ureteral stent erosion, disappeared following aspiration.

## Case presentation

**Fig. 1:**
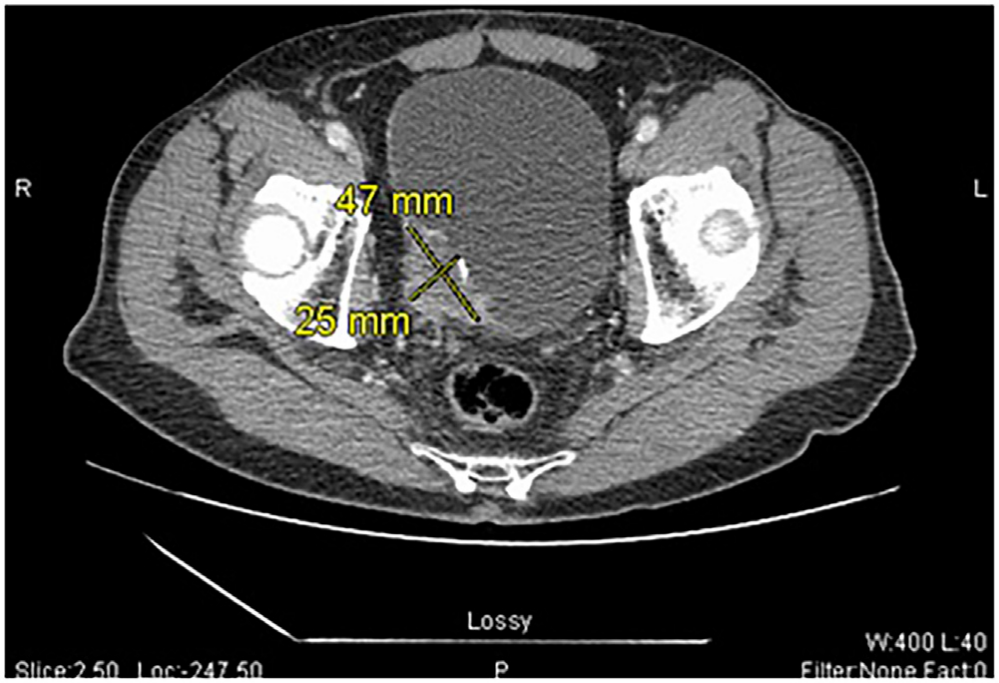
Axial computed tomography (CT) views of the incidental 2.5 × 4.7 cm mass. The mass is located in the right, posterior bladder, and has irregular margins with nonhomogeneous soft-tissue density.

**Fig. 2:**
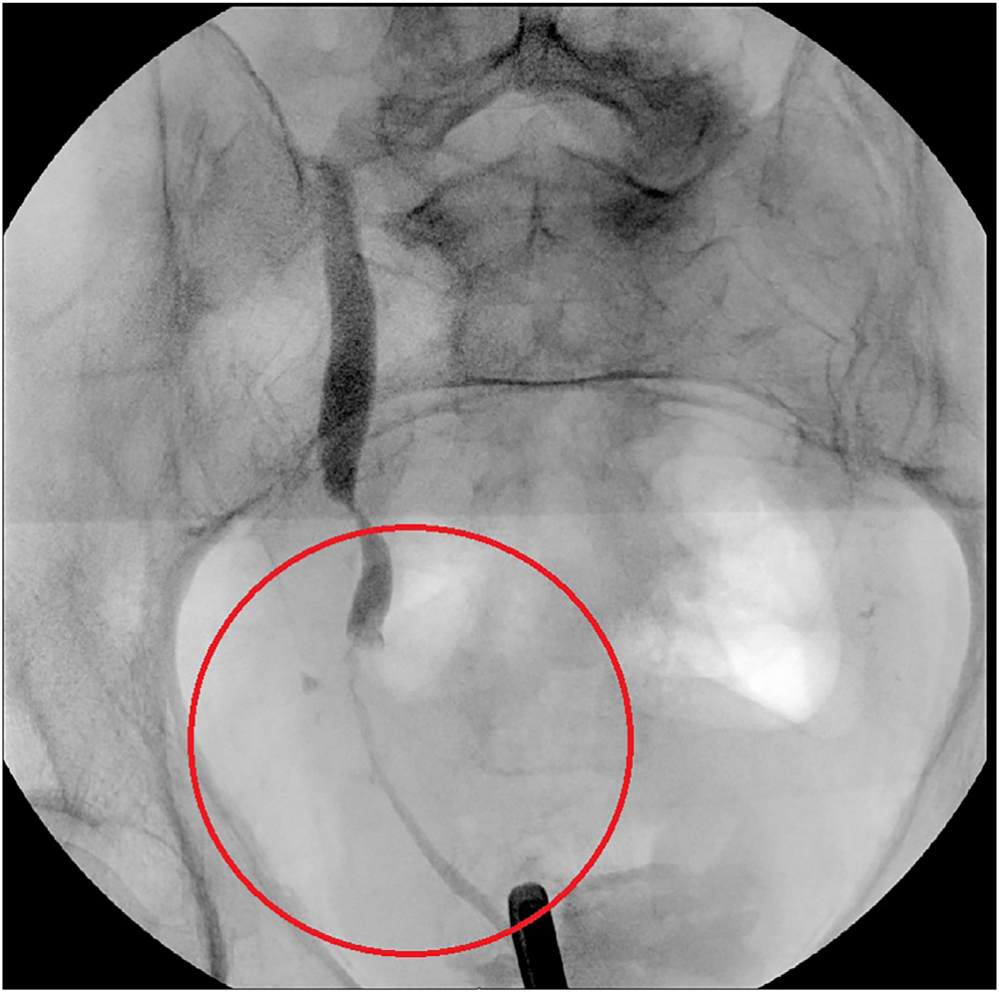
Fluoroscopic image of distal ureter stenosis. The narrowed ureter is due to malignant obstruction or possible ureteral stricture.

The patient is a 79-year-old male presenting to the Emergency Department (ED) for evaluation of worsening dysuria and new onset hematuria. On admission, his wife reports that he has been experiencing generalized fatigue and weakness, in addition to his bladder symptoms. The patient has a history of colon adenocarcinoma and benign prostatic hyperplasia (BPH). His surgical history includes extensive chemoradiotherapy prior to gross colon resection for colonic carcinoma over 20 years ago. The patient’s family reported no significant changes except for some weight loss over the last few months. He appeared lethargic and, other than nondescriptive abdominal pain, the patient denied any recent history of congestion, sore throat, cough, shortness of breath, nausea/vomiting, or diarrhea. Lab work and vital signs were unremarkable, and the patient was less likely to have an infectious etiology. Urology was consulted for management regarding a possible obstruction seen on ultrasound and secondary hydronephrosis. Computed Tomography (CT) Scan of the abdomen revealed an incidental, obstructive mass in the posterior bladder (Fig. 1). Fluoroscopy showed significant obstruction and stenosis of the distal right ureter (Fig. 2), and cystoscopy biopsy later revealed irregular cytology consistent with urothelial (Transitional cell) carcinoma. Patient was discharged with follow-up through urology for primary management after decompression was performed with a 6 French, 24 cm Ureteral stent. Eventually, the patient received a noncomplicated Transurethral resection of the bladder tumor (TURBT) in the following month.

**Fig. 3:**
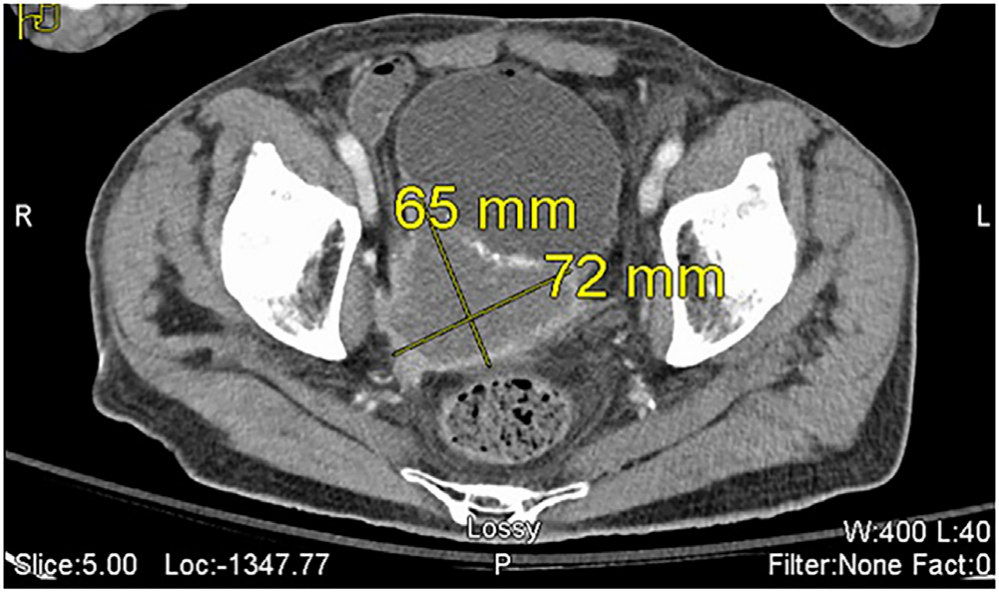
Axial CT views of the urothelial carcinoma 4 months after transurethral resection. The irregular mass has more than doubled in size to 65 × 72 mm and is in the same region as the previously resected urothelial cancer, suggesting likely recurrence.

Four months later, the patient returned to the hospital with another episode of abdominal pain and right flank pain. On readmission, the patient’s family stated that he had been having consistent urinary difficulties in addition to new-onset fever and chills that began 2 days ago. Vital signs on admission additionally revealed tachycardia with hypotension. With concerns for sepsis, initial laboratory workup, including Complete Blood Count (CBC) and Comprehensive Metabolic Panels (CMP), revealed hypokalemia (2.7 mmol/L), and elevated creatinine (1.61 mg/dL). Leukocytosis was evident with an elevated White Blood Cell (WBC) count of 18.3 K/uL and 92% neutrophilic predominance. Hemoglobin was 10.9 g/dL, and INR was 1.42. In addition to routine lab work. Computed tomography (CT) imaging was ordered by the hospital team in hopes of finding an occult abscess or a source of infection. CT imaging of the abdomen revealed diffuse bladder wall thickening, and a 65 × 72 mm irregular mass in the right posterior aspect of the bladder consistent with recurrent urothelial cancer. There was significant obstruction of the right ureterovesicular junction (Fig. 3).

**Fig. 4:**
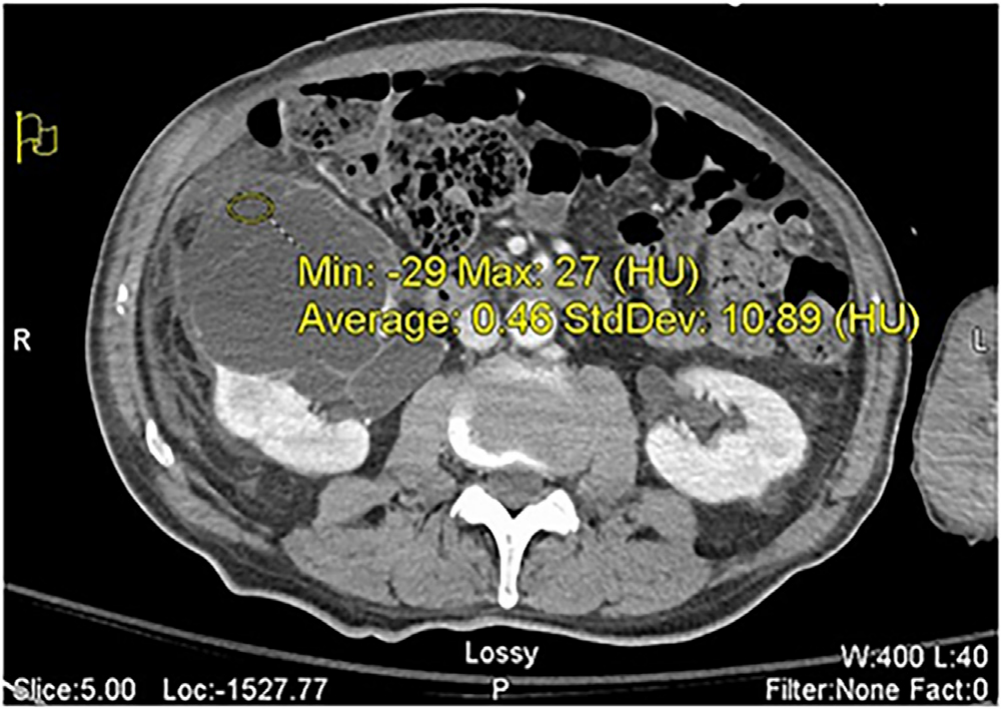
Axial views of the proximal ureter hydronephrosis and urinoma. The ill-defined margins of the renal cyst-urinoma collection are from a collecting system rupture. Hounsfield (HU) density of the fluid is within the expected range of simple, cystic fluid and urine.

Additionally, a large right renal cyst was present, consistent with the location of the patient’s reported right flank pain. There was significant right-sided hydroureteronephrosis and urinoma formation from the ruptured collecting system. The rupture and large urinoma required immediate decompression from interventional radiology (IR) with a percutaneous nephrostomy tube (Fig. 4).

**Fig. 5:**
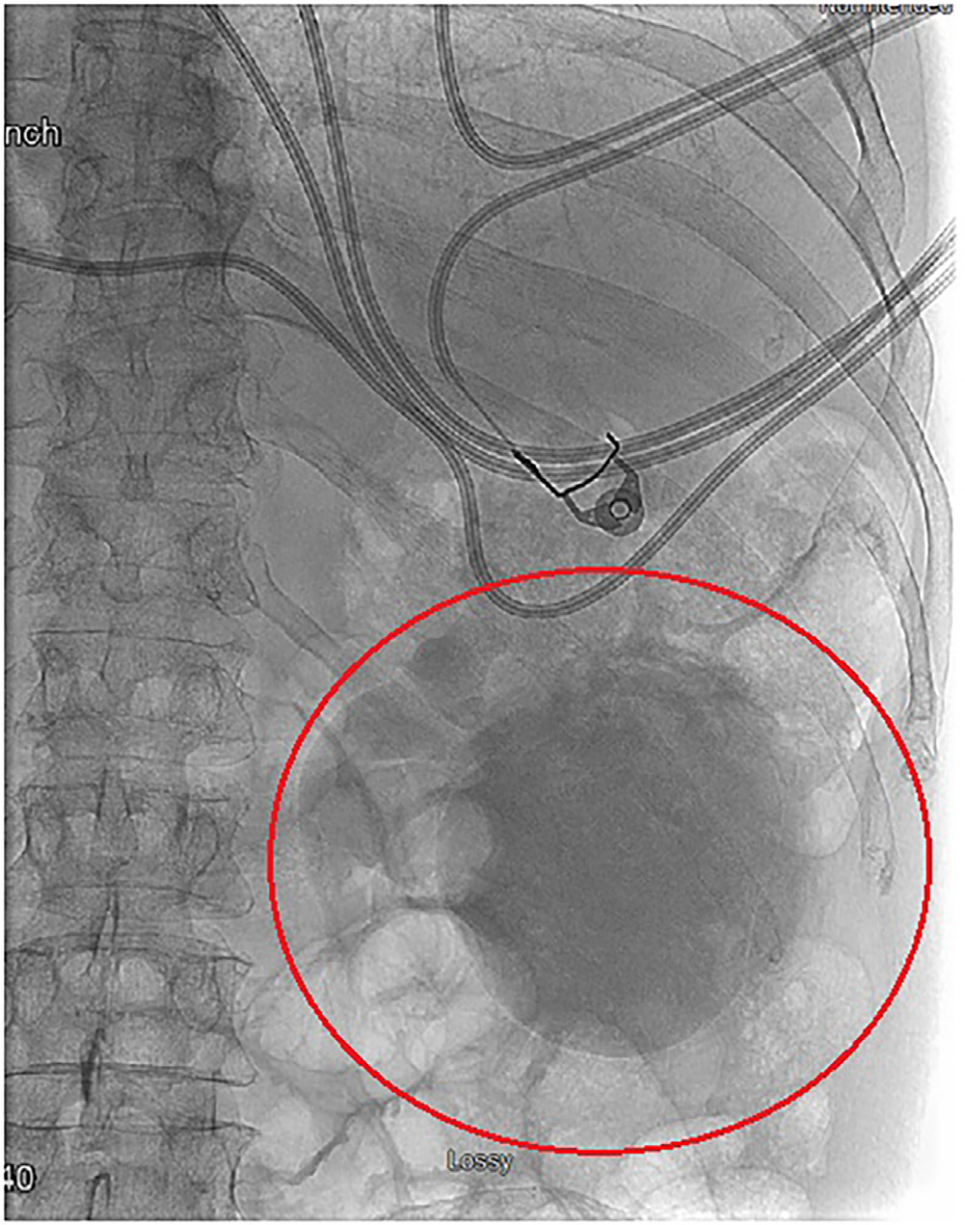
Contrast collection in the renal cyst of the anterior-lateral aspect of the right kidney. The contrast collection is due to a connection with the right renal calyx. The patient is prone in this image.

**Fig. 6:**
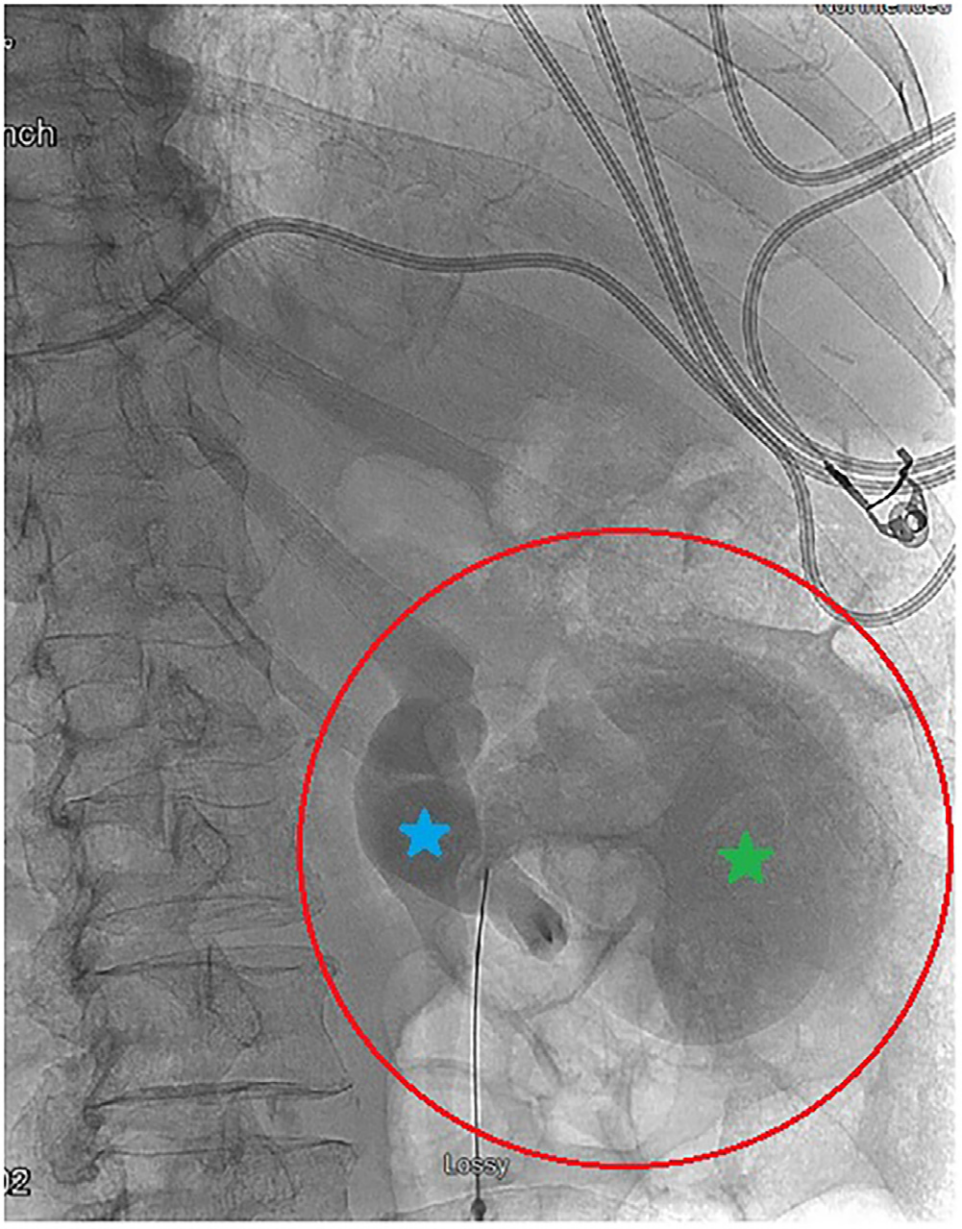
Fluoroscopic imaging of the contrast collection in the right renal cyst. Contrast from the earlier CT imaging that day has collected in the renal cyst. This implies a communication between the renal cyst and the collecting system’s renal pelvis (blue).

During the IR nephrostomy procedure, fluoroscopic imaging revealed the large, right renal cyst now filled with the contrast collection from the day’s prior CT scan (Fig. 5). Because contrast was clearly seen in the renal cyst, there was an evident communication noted between the renal cyst and the collecting system (Fig. 6).

**Fig. 7:**
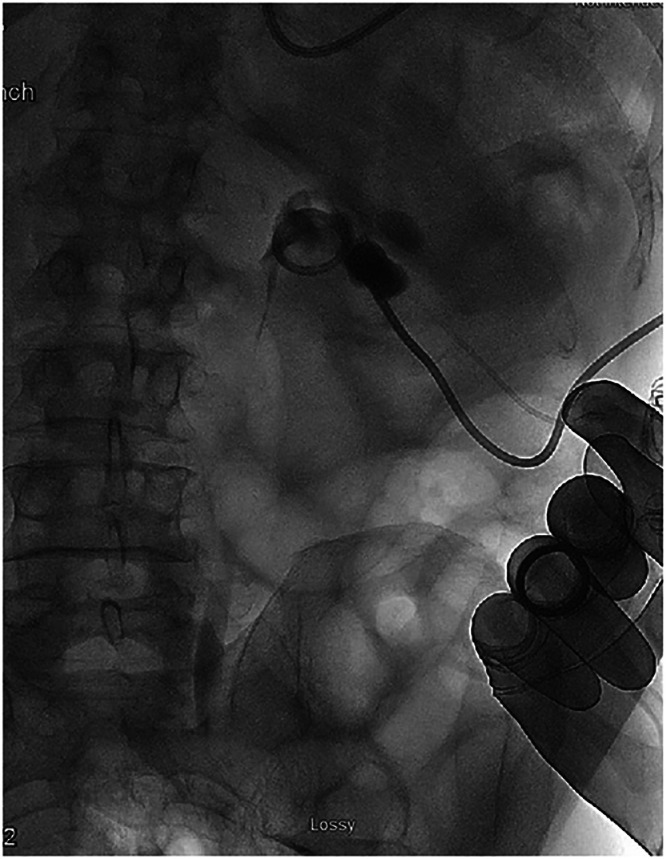
Fluoroscopic aspiration of the right renal cyst. A 5 French Yueh needle was advanced into the contrast collection of the cyst, and the cystic contents were aspirated completely. No residual contrast is seen outside of the collecting system or in the renal cyst.

**Fig. 8:**
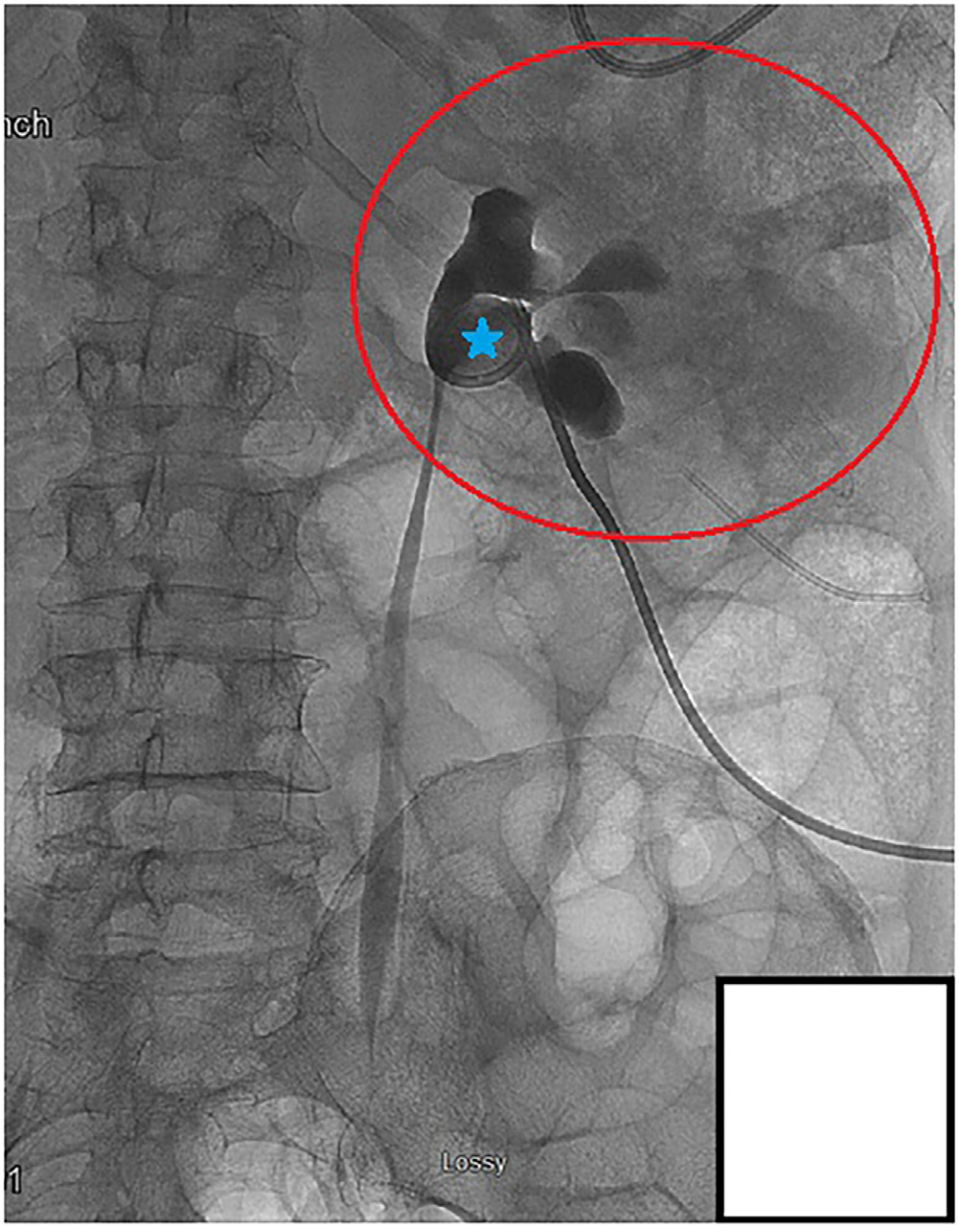
Regression of collecting system communication with the renal cyst. Fluoroscopic guided contrast injection into the renal pelvis (blue) shows the patent architecture of the major calyces, but no communication or renal cyst uptake of the contrast agent is seen. The once visible communication between the collecting system and the cyst is no longer present.

Decompression of the collecting system was achieved through an 8 French nephrostomy tube. Aspiration of the cystic contents was performed under fluoroscopic guidance with successful decompression of the renal cyst with no residual contrast present outside of the collecting system. Following decompression, contrast was injected into the nephrostomy tube, which demonstrated no extravasation from the collecting system and no communication with the cyst (Fig. 7). To confirm that there was no longer an active communication after the cyst decompression, additional fluoroscopic-guided contrast was injected into the renal pelvis with no visible flow into the renal cyst (Fig. 8).

Sclerotherapy with liquid sclerosant was initially deferred due to the communication between the renal cyst and collecting system, but was considered at this point to prevent re-expansion of the cyst. The IR attending chose not to proceed with cyst sclerotherapy due to the resolved communication following aspiration. There were no postprocedure complications. In addition to his antibiotic regimen with vancomycin and cefepime, the patient’s fever subsided, and he promptly returned to a stable baseline within 5 days of the procedure. After the 5th day following the procedure, a discussion with the family and goals of care were initiated given the recurrence of his bladder cancer and metastatic disease.

He was discharged to hospice where he died on day 3 following his discharge.

## Discussion

Renal cysts are typically acquired spontaneously or inherited from dysplastic changes to the renal tubular cilia that occur at the medullary-cortex interface in response to chronic ischemia or obstruction [8]. In the case of our patient, the initial renal cyst that developed was likely a result of chronic hydroureteronephrosis (Fig. 4) secondary to his urothelial carcinoma. Given that extrinsic compression of the urinary tract by malignancies can lead to severe hydronephrosis and acute kidney injury, the hydronephrosis likely contributed to the increased reno-pelvic pressure conditions favorable for cystic growth [9].

In the case of our patient, contrast uptake, which was present in the large, right renal cyst, indicated an evident communication between the renal cyst and the collecting system as normal contrast drainage should proceed distally through the path of least resistance, into the bladder (Fig. 5, Fig. 6). This communication is typically rare given that most renal cysts are in the superficial renal parenchyma [8]. Though communication between the collecting system and renal cysts has been noted within the literature, the mechanism behind the formation of renal cyst communications is not fully understood. One case report found a communicating cyst that occurred possibly due to increased renal pelvic pressure from hydronephrosis or distal obstruction due to a renal stone [10]. Rupture of the upper urinary tract is typically caused by renal stones and is much less frequently seen in the setting of urothelial malignancies [11]. A rare instance of a proximal ureter rupture in the setting of urothelial carcinoma was reported in a case of a 62-year-old patient and was ultimately attributed to a spontaneous rupture [11]. Though it is possible a similar spontaneous rupture occurred in our patient, we propose that it was more likely that the increased renal pelvic pressure from hydroureteronephrosis secondary to urothelial carcinoma led to rupture and subsequent urinoma formation (Fig. 4) and communication between the renal cyst and collecting system. This mechanism may help us bridge the gap between the rare phenomenon of proximal ureter rupture in the setting of malignancy and the development of communicating renal cysts.

Typically, renal cyst communications partially seal, and communication with the collecting system is maintained, which allows the cyst to regrow in the future [12]. This, however, did not occur in our patient as when additional contrast was injected postaspiration, the clear contrast communication was not reproduced on repeat fluoroscopy (Fig. 7, Fig. 8). The communication between the collecting system and the cyst disappeared. It is unclear why this occurred and there is very limited literature exploring the general regression of renal cyst communications. On their own, noncommunicating renal cysts tend to increase in size over time, with spontaneous regression being extremely rare [13]. Though it is possible spontaneous regression may have led to closure of the communication, it is less likely in our patient due to the established communication and how fast it closed in our patient following aspiration. Rupture of hemorrhagic renal cysts with internal hemorrhage can lead to partial decompression of the cyst and subsequent shrinkage of the residual cavity [14]. Additionally, infected renal cysts have been shown to partially collapse following drainage, often leaving behind a thickened or “bunched-up” cyst wall [15]. We propose that a similar decompression mechanism occurred in our patient, and that the increased renal pelvic pressure from the hydroureteronephrosis may have been responsible for keeping the initial communication open. Once the nephrostomy tube was in place and the pressure was significantly reduced, it is possible that the pressure gradient disappeared.

This unique disappearance of the communication between the renal cyst and communicating system and the mechanism behind it poses a potential future direction of research and may ultimately contribute to a better understanding of management of these rare communications given that communications only typically partially seal. Current treatment of renal cysts include aspiration and sclerotherapy or laparoscopic surgery to remove the cyst [1]. However, sclerosing agents are contraindicated in patients with renal cyst communications due to the potential for spillage of the sclerosant into the collecting system or retroperitoneum [12]. There is limited evidence regarding management of communicating renal cysts, though one case report performed successful renal sparing surgery in a communicating hydatid cyst [16].

### Limitations

One limitation of this study is that follow-up imaging could not be acquired as the patient died 3 days after being discharged to home hospice care. Further contrast-enhanced CT scans could have provided clearer evidence into the cause of the regression of the communication. Furthermore, renal cysts can share similar densities as dilated ureters in the setting of hydronephrosis [17]. Typically, renal cysts do not uptake contrast enhancement in CT scan studies because they are located superficially in the renal cortex, furthest away from the collecting system [17]. With this said, when communication occurs between cysts and the pyelocaliceal system, further distinctions on contrast-enhanced CT may be needed in the future to better distinguish between renal cysts and hydronephrosis as this defining feature of simple renal cysts is no longer identifiable.

## Conclusion

In conclusion, renal cyst communications to the collecting system are exceedingly rare, but are important to establish, especially in the management of renal cyst aspirations. We propose that a pressure-like mechanism secondary to hydroureteronephrosis in the setting of urothelial carcinoma contributed to the development of the communicating renal cyst itself. Unique to our patient, the immediate closure of the communication following decompression is different from the typical presentation of renal cyst communications. Though the exact etiology and resolution of the cyst could not be precisely determined due to limitations in follow-up imaging, this case report and its abnormal presentation adds to the growing body of literature relating to renal cysts communications.

## Patient consent

The patient provided written informed consent for their clinical information to be used in this case report and was informed that any identifying information would be anonymized. The patient understands that this case report may be published and will be used for educational and research purposes. Patient-signed consent form can be submitted after patient health information is redacted upon request from reviewer/editor.
